# Flying to high-altitude destinations: Is the risk of acute mountain sickness greater?

**DOI:** 10.1093/jtm/taad011

**Published:** 2023-01-25

**Authors:** Johannes Burtscher, Erik R Swenson, Peter H Hackett, Grégoire P Millet, Martin Burtscher

**Affiliations:** Institute of Sport Sciences, University of Lausanne, Lausanne 1015, Switzerland; Department of Biomedical Sciences, University of Lausanne, Lausanne 1005, Switzerland; VA Puget Health Care System, University of Washington, Seattle, WA, USA; Altitude Research Center, Division of Pulmonary Sciences and Critical Care Medicine, Department of Medicine, University of Colorado Anschutz Medical Campus, Aurora, CO, USA; Institute of Sport Sciences, University of Lausanne, Lausanne 1015, Switzerland; Department of Biomedical Sciences, University of Lausanne, Lausanne 1005, Switzerland; Department of Sport Science, University of Innsbruck, Innsbruck A-6020, Austria; Austrian Society for Alpine and High-Altitude Medicine, Innsbruck A-6020, Austria

**Keywords:** Air travel, high altitude, hypoxia, acute mountain sickness

## Abstract

**Background:**

Altitude sojourns increasingly attract individuals of all ages and different health statuses due to the appeal of high-altitude destinations worldwide and easy access to air travel. The risk of acute mountain sickness (AMS) when flying to high-altitude destinations remains underemphasized. Thus, this review aims to evaluate the altitude-dependent AMS incidence depending on the mode of ascending, e.g. by air vs terrestrial travel.

**Methods:**

A literature search was performed to identify the observational studies assessing AMS incidence after acute ascent of primarily healthy adults to real high altitude. In addition, placebo arms of interventional trials evaluating the prophylactic efficacy of various drugs have been separately analysed to confirm or refute the findings from the observational studies. Linear regression analyses were used to evaluate the altitude-dependent AMS incidence.

**Results:**

Findings of 12 observational studies, in which the AMS incidence in 11 021 individuals ascending to 19 different altitudes (2200–4559 m) was evaluated, revealed an impressive 4.5-fold steeper increase in the AMS incidence for air travel as compared with slower ascent modes, i.e. hiking or combined car and/or air travel and hiking. The higher AMS incidence following transportation by flight vs slower means was also confirmed in placebo-treated participants in 10 studies of drug prophylaxis against AMS.

**Conclusions:**

Due to the short time span in going from low to high altitude, reduced acclimatization likely is the main reason for a higher AMS risk when travelling to high-altitude destinations by flight. To avoid frustrating travel experiences and health risks, appropriate and timely medical advice on how to prepare for air travel to high altitude is of vital importance. Effective preparation options include the use of modern pre-acclimatization strategies and pharmacological prophylaxis by acetazolamide or dexamethasone, or even considering alternate itineraries with more gradual ascent.

## Graphical Abstract

**Graphical Abstract:**
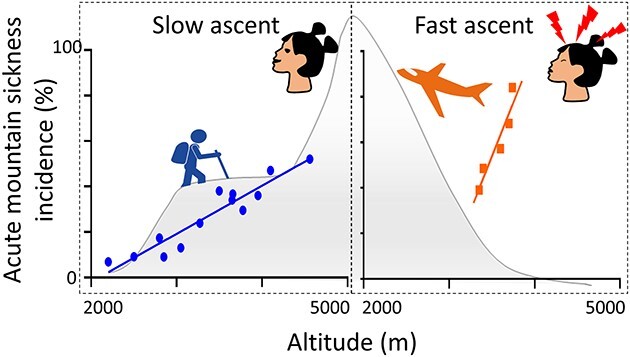

## Introduction

High-altitude destinations all over the world (e.g. La Paz, 3640 m, Bolivia; Leh, 3500 m, Ladakh; Cusco, 3399 m, Peru; Lhasa, 3356 m, Tibet) are attracting millions of travellers and pilgrims every year. Reasons to travel include sightseeing and business activities, trekking or high-altitude climbing, study purposes or visiting relatives and friends to benefit from low allergen concentrations or to escape from the heat waves of low-altitude areas. People of all ages and both sexes, of a broad range of fitness and varying health conditions, expose themselves to high-altitude environments and are differentially affected based on individual characteristics and vulnerabilities.^1–3^ The decreasing availability of oxygen with increasing altitude can trigger the development of high-altitude illnesses (HAIs), i.e. AMS, high-altitude cerebral (HACE) and pulmonary oedema (HAPE).^4–7^ Besides individual susceptibility, the speed of ascent also determines the risk for such adverse altitude effects.^3^^,^^4^^,^^7^ AMS is by far the most prevalent HAI, affecting ~17% of all individuals who rapidly ascend to 2800 m^8^ and >50% if the target altitude is >4500 m.^9^ Shlim recently emphasized about the elevated risk for HAIs by rapid travel to high-altitude destinations and about the potential benefits of prophylactic drug use.^10^ Although >25 years ago, Murdoch *et al*. referred to an extraordinarily high AMS risk of 84% when rapidly ascending to high altitudes (3740 m) by airplane,^11^ whether fast passive (i.e. by airplane or car) or slower active (i.e. by hiking), ascents that are associated with a higher incidence of AMS requires further assessment. This factor, however, is of high clinical relevance for the huge numbers of air travellers visiting high-altitude destinations. In the present review, we therefore aimed to evaluate the altitude-dependent AMS incidence depending on the mode of ascending.

## Methods

We searched the literature (PubMed and Web of Science) for original (observational) studies assessing the AMS incidence after acute ascent (1–3 days) to terrestrial high altitude. Search terms used were ‘acute mountain sickness’ and ‘incidence OR prevalence’. Studies with the main objective to evaluate altitude-dependent AMS in unacclimatized but primarily healthy adults, aged ≥18 years, were included. Additional inclusion criteria were that the AMS diagnosis was made within the first 24 hours after arrival at altitude and was based on at least three AMS symptoms^12^^,^^13^ or a Lake Louis Questionnaire Score (LLQS) of 3 or more.^14^^,^^15^ Studies were categorized according to the type of ascent, i.e. (i) passive (travel by plane or car), active (travel by foot) ascents or both and (ii) very rapid (1 day) and rapid (2–3 days) ascents. Studies on pilgrims and those without clear information on the type of ascent, or whether prophylaxis (by specific pre-acclimatization strategies or pharmacological interventions) was applied, were excluded. Linear regression analyses were used to evaluate the altitude-dependent AMS incidence and an extra sum of squares *F*-test was applied to compare the slopes of the regression models using Graphpad Prism, version 9.

In order to further confirm or refute the findings derived from these observational non-intervention epidemiologic studies, we also separately analysed the placebo arms of a number of studies evaluating the prophylactic efficacy of various drugs (i.e. acetazolamide, corticosteroids and non-steroidal anti-inflammatory drugs) during rapid (1–2 days) ascents to terrestrial high altitude. Owing to the small sample sizes and the possibility of placebo and nocebo effects in these types of interventional studies,^16^ we did not include them in our primary analysis.

**Table 1:** AMS incidence recorded at different altitudes depending on the type of ascent

Reference	Altitude (m)	*N* total	Male (m), female (f)	AMS + (%) LLQS ≥3^a^	AMS + (%) LLQS ≥4	Ascent in 1 (1) or 2–3 (2) days	Ascent by foot (1), car (2), airplane (3)	AMS + age difference^b^ Yes = 1, no = 2	AMS + sex difference^b^ Yes =1, no =2
Maggiorini *et al*., 1990^13^	2850–4559	466	83% m, 17% f	34^a^		1 + 2	1	1 (<20 and >40)	2
	2850	47		9^a^		1	1		
	3050	128		13^a^		1	1		
	3650	82		34^a^		1	1		
	4559	209		52^a^		2	1		
Murdoch *et al*., 1995^11^	3740	116	55% m, 45% f	84		1	3	NA	1, female
Mairer *et al*., 2009^21^	2200–3500	431	76% m, 24% f		16.2	1	1	2	2
	2200	159			6.9	1	1		
	2500	55			9.1	1	1		
	2800	138			17.4	1	1		
	3500	79			38	1	1		
Ren *et al*., 2010^17^	3600	3628	>99% m	57.2		1	3	NA, only young people included	NA
Wang *et al*., 2010^22^	3952	1066	67% m, 33% f	36	28	2	1	1, young	2
Salazar *et al*., 2012^18^	3400	991	44.5% m, 55.5% f	48.5		1	3	1, young	2
Gonggalanzi *et al*., 2016^23^	3658	2385	55% m, 45% f		36.7	1 + 2	2 + 3	1, young	2
Horiuchi *et al*. 2016^24^	3776	345	59% m, 41% f	29.5		2	1	2	2
Shen *et al*., 2020^25^	4100	99	71% m, 29% f	47		2	1	2	1, female
Yang *et al*., 2020^26^	3272	345	62.4% m, 37.6% f	23.9		1	1	1, young	2
Caravedo *et al*., 2021^19^	3350	123	43% m, 57% f	39		1	3	2	1, female
Chen *et al*., 2021^20^	3700	1026	100% m	68.3		1	3	NA	NA

NA, not assessed.

a Three or more AMS symptoms indicative of AMS.^12^

b The most affected populations are indicated.

## Results

**Figure 1:**
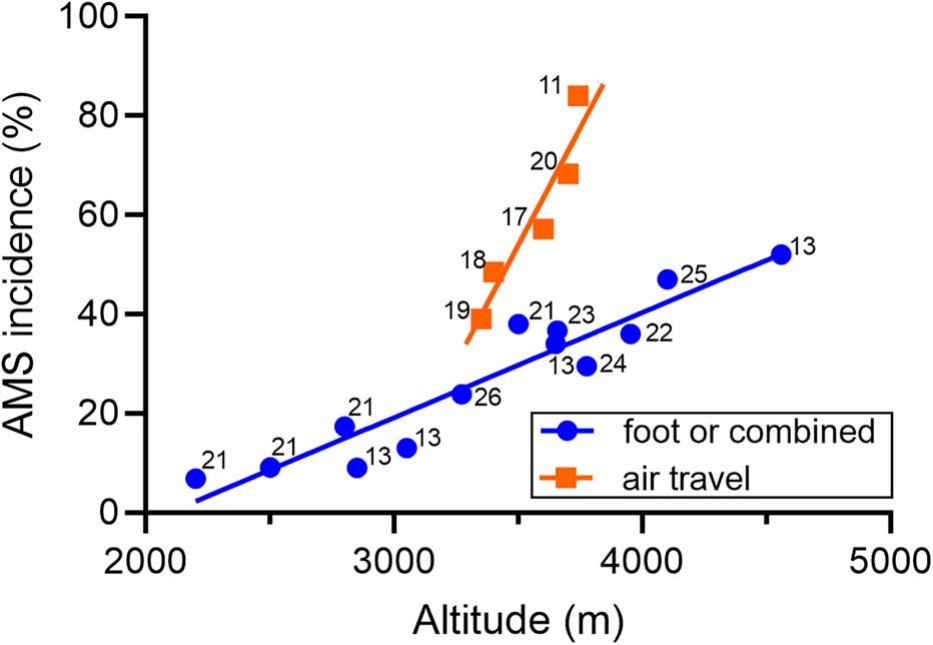
Altitude-dependent AMS incidence when travelling to high altitude by airplane or by foot and/or by car and plane; *R*^2^ for both regressions is 0.9 and the slopes are significantly different (*P* < 0.01), as determined by an extra sum of squares *F*-test (*F*_(1, 14)_ = 21.90); numbers in the graph indicate references to the respective publications.

We analysed a total of 12 studies in which the AMS incidence in 11 021 individuals ascending to 19 different altitudes (2200–4559 m) was evaluated. The findings of five studies of participants,^11^^,^^17–20^ who predominantly used air travel to high-altitude destinations, were compared with those of seven studies of travellers who primarily ascended by foot and/or car and plane.^13^^,^^21–26^ The characteristics of the selected studies are shown in Table 1. Overall, more study participants were male than female. While AMS scoring in one study was based on the presence of three AMS symptoms (before the availability of the LLQS), the other studies considered a LLQS of ≥3 (eight studies) or of ≥4 (three studies) as indicative for AMS. Young people and females seem to be more frequently affected by AMS than older individuals and men (Table 1). The AMS incidence further increased linearly with altitude, and this increase was significantly 4.5-fold steeper when ascending (rapidly) by air travel (slope = 0.094) compared with slower ascents by foot or a combination of air travel, car and foot (slope = 0.021) (Figure 1).

**Figure 2:**
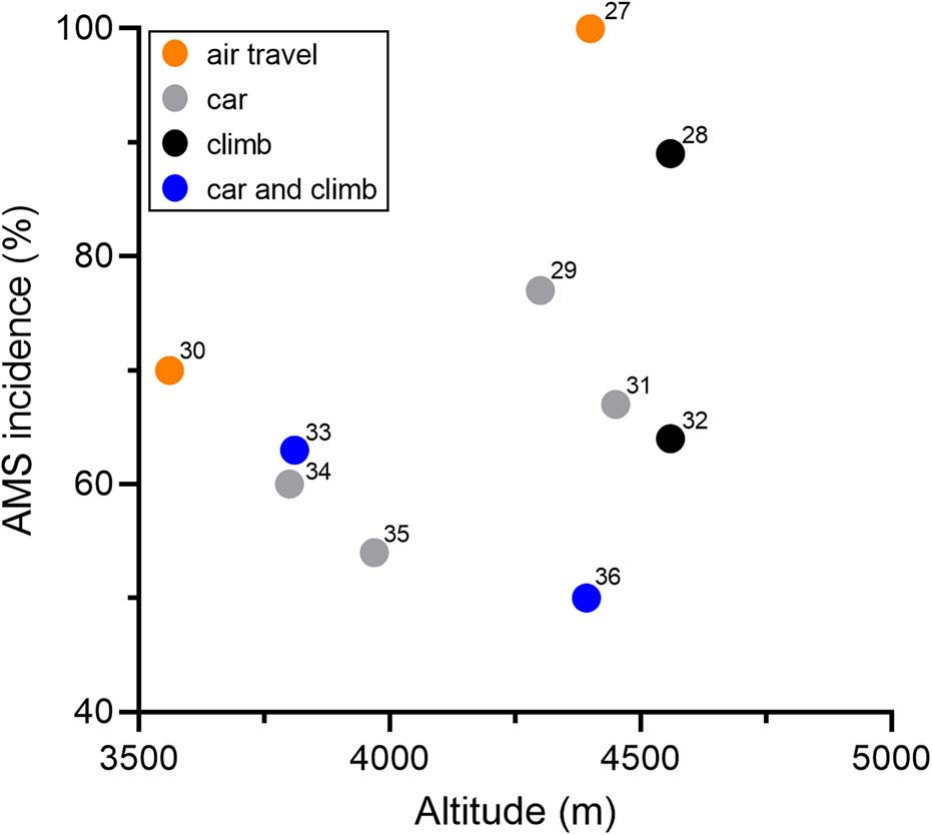
AMS incidence, speed of ascent and the final altitude reached in participants of the placebo arms of interventional studies evaluating various types of pharmacological AMS prophylaxis; numbers in the graph indicate references to the respective publications.

The AMS incidence reported in participants of the placebo arms of interventional studies evaluating various types of pharmacological AMS prophylaxis are shown in Table 2. For reasons of comparability (with Figure 1), the AMS incidence depending on the final altitude and mode of travel are depicted in Figure 2.

**Table 2:** AMS incidence, mode and speed of ascent and the final altitude reached in participants of the placebo arm of interventional studies evaluating various types of pharmacological AMS prophylaxis

Reference	Altitude (m)	Mode (and speed) of ascent	*N*, placebo arm; age; sex (% male)	AMS + (%)	Remarks
Hackett *et al*., 1988^27^	4400	Air travel (1 hour)	8; 28 ± 1; 100	100	Travel by helicopter
Maggiorini *et al*., 2006^28^	4559	Climb (1 day)	9; 41 ± 8; 78	89	HAPE susceptibles
van Patot *et al*., 2008^29^	4300	Car (2 hours)	22; 24 ± 6; 43	77	Staging at 2000 m
Wang *et al*., 2013^30^	3561	Air travel (3 hours)	10; 25 ± 2; 100	70	
Burki *et al*., 1992^31^	4450	Car (8 hours)	6; 20 ± 1; 100	67	
Parati *et al*., 2013^32^	4559	Climb (1 day)	22; 37 ± 10; 50	64	
Lipman *et al*., 2018^33^	3810	Car, climb (1 day)	35; 32 ± 7; 40	63	
Chow *et al*., 2005^34^	3800	Car (2 hours)	20; 34 ± 10; 50	60	Overnight stay at 1230 m
Moraga *et al*., 2007^35^	3969	Car (8.5 hours)	12; 22 ± 1; 100	54	
Ellsworth *et al*., 1991^36^	4392	Car, climb (2 days)	18; 33 ± 4; 75	50	

Ten studies, including 162 participants (placebo arms), report an AMS incidence between 50 and 100% when rapidly (1 hour–2 days) ascending to altitudes between 3651 and 4559 m. Not surprisingly, the number of participants of the placebo arms in these clinical trials is much lower compared with that of the observational studies shown in Table 1. Only two trials evaluated the AMS incidence after air travel to high altitude, but incidence data fit well with those presented in Figure 1, i.e. air travel (helicopter) within 1 hour to 4400 m was associated with an AMS incidence of 100%^27^ and that to 3561 m within 3 hours was associated with an AMS incidence of 70%.^30^ However, similarly, high AMS incidence was observed after rapid ascent (within a few hours) by car and/or strenuous climbing to altitudes >4000 m,^29^ particularly in susceptible individuals, e.g. those prone to suffer from HAPE.^28^ The remaining six studies report an average AMS incidence of 60% when arriving at an average altitude of 4163 m.^31–36^ This is higher than the AMS incidence observed in the non-flight studies but clearly lower than that of the observational air travel studies (Table 1, Figure 1).

## Discussion

The present findings, derived from mostly large observational studies, indicate an impressive 4.5-fold steeper increase in AMS incidence when quickly ascending to high-altitude destinations by air travel, i.e. from 39% at 3350 m to 84% at 3740 m (~11.5% per 100 m gain in altitude). By contrast, slower ascent by foot or combined walking with car and air travel was associated with an only 2.1% increase in AMS incidence per 100 m (Figure 1). Younger people and females suffer more frequently from AMS. Despite earlier reports of the high risk of developing AMS^11^ or headache^37^ when flying to high altitude, the remarkable differences between the AMS incidence when travelling to high altitude by airplane compared with slower ascent modes have previously been underemphasized. Travellers flying to high-altitude destinations are thus mostly not aware of the higher AMS risk they may experience.

The increasing accessibility of appealing high-altitude destinations worldwide by air travel^1^ contributes to growing numbers of high-altitude sojourners and reduces age- and health-related barriers. The insufficiently understood interactions of chronic illnesses and age with HAIs add to the clinical relevance of these developments.

Although the risk of altitude illness is higher when flying, health consequences may be less severe compared with trekking or climbing, likely due to better access to medical care in the cities. Still, flying to high altitude remains a considerable health risk, as confirmed, e.g. by the study of Ren and colleagues, who reported that 12% of air travellers needed hospitalization and 2% suffered from HAPE when flying to an altitude of 3600 m.^17^ Moreover, the risk of HAIs increases considerably in subjects who continue with trekking/climbing too fast after arrival by air travel.^12^

The lack or minimal acclimatization to high altitude (hypoxia) due to the very short time span from low to high altitude likely is the main reason for the high AMS risk when flying to high altitude, which is supported by the demonstrated short-term benefits of high-altitude acclimatization.^38^^,^^39^ Cabin air pressure of commercial flights corresponds to an altitude of 1981–2438 m (6500–8000 ft) and is associated with a respective decrease in arterial oxygen saturation (from 97% at sea level to ~92.5% at 2438 m).^40^ These hypoxemic levels are usually well tolerated by healthy passengers and lead to AMS development in <10% of passengers.^40^ In-flight hypoxia may even initiate acclimatization processes, but those are not sufficient when landing at high altitudes with a considerably lower atmospheric pressure. While this remains to be evaluated, it is conceivable that the symptoms of general discomfort developing during the flight,^40^ and/or jet lag following long haul travel, might even adversely affect the acclimatization process and aggravate the AMS risk.^41^

The slightly higher AMS incidence observed in the placebo-treated participants of non-flight studies (Table 2, Figure 2) might also be the result of a more rapid ascent to higher altitudes which is associated with the study participants being unable to customize their ascent speed due to the protocols that require equal ascent rates as of those on AMS prophylaxis. Slower and individually tailored ascent rates are certainly the main reason explaining the much lower AMS incidence, (i.e. 20–34% in those without pharmacological prophylaxis) during many-day high-altitude treks.^42–45^ Kayser and colleagues nicely demonstrated the steep increase of AMS incidence from 34 to 86% in mountaineers hiking slowly (average = 14 m/hour) to 5896 m when compared with acute exposure to the same simulated altitude.^46^ The impact of rapid ascent from 2000 to 4500 m has also been convincingly quantified by Beidleman and colleagues based on observations from 20 studies (10 at real and 10 at simulated altitude).^47^ These authors found an almost linear increase of AMS incidence (from <10% to >80%) in this large cohort (*N* = 308) of young and middle-aged (18–45 years) unacclimatized but fit males and females, who rapidly ascended (<2 hours) from ~2000–4500 m.^47^ Importantly, AMS severity peaked between 18 and 22 hours of exposure and was (in these studies) more pronounced in males and aggravated by physical activity but returned to baseline (regardless of sex and physical activity) after around 48 hours.^47^ In the studies of rapid active ascents, these all entailed physical exertion, which is known to increase the AMS severity, likely by the greater degree of hypoxemia over that at rest that occurs with exercise at high altitude.^47^^,^^48^ This is in addition to the lack of time to physiologically acclimatize by steadily increasing ventilation, a process that starts immediately and becomes complete after several days at high altitude.^49^ Although AMS is usually benign and self-limited (disappears during the first days at altitude), in rare cases, it may progress to or be accompanied by life-threatening HACE or HAPE.^3–7^ In addition, AMS symptoms (i.e. headache, nausea, vomiting, dizziness and fatigue) may by themselves curtail otherwise pleasant experiences during high-altitude sojourns, such as enjoying food, sleep, landscape, sightseeing and other physical activities. For rather short stays at high altitude, AMS can be responsible for not only adverse health effects but also a frustrating travel experience.

Therefore, appropriate and timely medical advice on how to prepare for air travel to high altitude is of vital importance. Such preparation includes pre-acclimatization and pharmacological prophylaxis^1^^,^^50^^,^^51^ and possibly suggesting alternate itineraries with more gradual ascent.

Pre-acclimatization strategies include both exposures to real altitude near home and/or normobaric hypoxia (hypoxia rooms and breathing hypoxic gas mixtures).^50–52^ Hiking and sleeping at altitudes >2500 m for at least 3 days close to the planned start of air travel and/or repeated exposures in hypoxia rooms (intermittently for at least 60 hours at a simulated altitude >2500 m) reduces the AMS risk associated with subsequent air travel to high altitude.^50^

Beside avoiding intense exercise during the first days at high altitude,^48^^,^^53^ the preventive use of acetazolamide supports acclimatization by increasing ventilatory drive to achieve better arterial oxygenation due to renal excretion of bicarbonate that counteracts the respiratory alkalosis.^3^^,^^10^^,^^54^ To achieve this effect, acetazolamide (125 mg bd) should be initiated 8–24 hours before ascent and should be continued for 48 hours at the final high-altitude destination.^55^ Travellers should further be advised not to ascend higher until mild symptoms have resolved and should descend or seek medical assistance when severe symptoms develop.^56^

Dexamethasone can be considered as an alternative to acetazolamide for adult travellers rapidly ascending by air. Although unlike acetazolamide, it does not facilitate acclimatization, studies show it to be very effective in preventing AMS with 2 mg every 6 hours or 4 mg every 12 hours, starting 4–6 hours before the ascent.^4^ Some experts recommend a higher dose of 4 mg every 6 hours for very rapid deployment over ~4000 m, such as for rescue work or military deployment that requires physical exertion.^57^ Owing to potential side effects (hyperglycaemia and mood changes), its use should be limited to no more than 5 days. It is most often used for 2–3 days, while natural acclimatization occurs (without facilitating it).

### Limitations

Although we tried to limit study inclusion on rapid ascent (1–3 days) to high altitude without essential pre-acclimatization and/or pharmacological prophylaxis, and the availability of clear information on the AMS incidence, confounding cannot entirely be excluded due to individual differences in pre-ascent, within-ascent and post-ascent conditions (e.g. chronic and/or acute diseases, physical activity patterns, dietary and sleeping habits) and differences in methods used for and interpretation of AMS diagnosis. However, the large study population of the observational studies and affirmation of the high AMS incidence after air travel in placebo arms of studies evaluating pharmacological AMS prophylaxis are the strengths of the present review.

## Conclusion

Visiting high-altitude destinations by air travel is associated with a higher risk of AMS development when compared with slower ascent by foot or combined travelling (walking, car and/or plane). To avoid frustrating travel experiences and health risks, appropriate and timely medical advice on how to prepare for air travel to high altitude is of vital importance. Besides the use of modern pre-acclimatization strategies, options of pharmacological prophylaxis or alternate itineraries with more gradual ascent should be considered.

## Funding

None.
